# The epidemiology of uveal melanoma in Germany: a nationwide report of incidence and survival between 2009 and 2015

**DOI:** 10.1007/s00417-021-05317-7

**Published:** 2021-10-04

**Authors:** Ahmad Samir Alfaar, Anas Saad, Peter Wiedemann, Matus Rehak

**Affiliations:** 1 Department of Ophthalmology, University Hospital Leipzig, University of Leipzig, Liebigstr. 10-14, 04103 Leipzig, Germany; 2 Experimental Ophthalmology, Charité–Universitätsmedizin Berlin, Berlin, Germany; 3 Heart and Vascular Institute, Cleveland Clinic Foundation, Cleveland, OH USA

**Keywords:** Melanoma, Uvea, Ciliary body, Choroid; incidence, Germany

## Abstract

**Purpose:**

To calculate the overall incidence of uveal melanoma in Germany and to compare incidences in different German states. In addition, we computed the overall and cancer-specific survival rates nationwide.

**Methods:**

Incidence data for the period between 2009 and 2015, covering the entire German population, was collected through the German Center for Cancer Registry. ICD-O-3 topography codes C69.3-C69.4 and histology codes for melanoma subtypes were used to collect the incidence data. Confidence Intervals with a level of 95% (95% CI) were calculated for rates. Survival was calculated using the Kaplan–Meier. The log-rank test was used for survival comparisons.

**Results:**

This study comprised 3654 patients with uveal melanomas, including 467 (12.8%) with iridial and ciliary body tumors. The overall age-standardized incidence rate (ASIR) was 6.41 person per million. Generally, the ASIR was higher in males than females (6.67 (95% CI 6.37–6.98) vs. 6.16 (95% CI 5.88–6.45 per million). Higher crude incidence rates were noted in the northeastern states (12.5 per million (95% CI 10.5–14.7) in Mecklenburg-Vorpommern) compared with the southwestern states (2.1 per million (95% CI 1.7–2.6) in Hessen). The 5-year overall survival stood at 47%, while the cancer-specific survival stood at 84%. Multivariate analysis showed that women, younger patients, and patients living in Berlin achieved significantly higher overall survival.

**Conclusion:**

Overall ASIR of uveal melanoma in Germany indicates that the disease is more common in males and that it follows the same geographical distribution previously noted in central European countries, with the highest incidence in northern parts of Germany. 
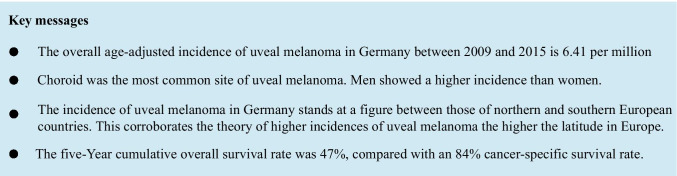

**Supplementary Information:**

The online version contains supplementary material available at 10.1007/s00417-021-05317-7.

## Introduction

Uveal melanoma is the most common adulthood primary ocular malignancy [1]. It most frequently arises from the choroid and less commonly from the ciliary body and iris. In the USA, the age-standardized incidence was estimated at 5.1 per million, a figure that remained constant over the years [2]. The disease rarely affects children [3]. The incidence increases with age and plateaus or declines after the age of 75 [2, 4]. Previous studies have shown a relation between incidence and era of birth, gender, ethnicity, and geographical location. White population and males were shown to have higher incidences of the disease [5]. Higher latitudes were associated with increased incidence of uveal melanoma in the USA [6]. A study conducted between 1983 and 1994 reported similar latitude-associated differences in the incidence of uveal melanoma, with incidences of 2 per million in southern Europe, compared with more than 8 per million in northern countries [4]. This study, however, examined data from only one German state, Saarland, which represents only 1% of the German population. Saarland is located on the very southwest borders with France and belonged to the previous West Germany, which had a different healthcare system than that of East Germany prior to unification in 1990 [7, 8]. Therefore, we expected to find a disparity in incidences of uveal melanoma and treatment outcomes between eastern and western states, including Saarland. We aimed at determining the crude and age-standardized incidence rates of uveal melanoma in Germany at the national level between 2009 and 2015. We further aimed to investigate disease characteristics and treatment outcomes including the nationwide overall and cancer-specific survival rates of uveal melanoma patients.

## Methods

### Study population and methods

Data from 2009 to 2015, covering the entire population of Germany, were gathered from the German population-based cancer registries through the German Center for Cancer Registry at the Robert Koch Institute together with the Association of Population Cancer Registries in Germany (GEKID) [9]. Data collected pertained to patients 15 years of age or older. Further details on the methodologies of the German Cancer Registry are available elsewhere [10].

Patients were identified as having uveal melanoma by using the ICD-O-3 topography codes, including choroid (ICD-O-3 topography code C69.3), the ciliary body (and iridial) code (C69.4), and histology codes for melanoma and malignant behavior codes. Patients initially coded as suffering from retinal melanomas (*n* = 10) were recoded as having choroidal disease. This was done with the knowledge of previous practices where there was often miscoding of this group of patients [2]. We used the International Classification of Diseases version 10 for the purposes of coding causes of death. Cause-specific survival rates were determined by analyzing deaths caused by choroid, ciliary body, and retinal disease (C693, C694, and C692, respectively). We excluded patients with unknown or benign disease behavior as well as those whose diagnoses were reported on death certificates only (DCO) to ensure quality of the data collected. No patients were reported as having had uveal melanoma based on DCO in this cohort. The TNM classification version 7 was used to determine staging in 88.1% of the tumors, followed by version 6 in 11.4%. Staging was determined in a minority of cases through the use of the TNM versions 8 (*n* = 13) or 5 (*n* = 4) [11]. For practical reasons, we have joined them together in the analysis.

Population estimates as well as the German Standard Population Report of the 2011 Census, both provided by the Federal Statistical Office, were used to calculate crude (CR) and age-standardized incidence rates (ASIR) [12]. We plotted the incidences of uveal melanomas in the federal states at annual intervals according to the annual population estimates provided by the Federal Statistical Office. For the purposes of this study, the German states of Schleswig–Holstein, Hamburg, Lower Saxony, Bremen, North Rhine-Westphalia, Berlin, Brandenburg, Mecklenburg-West Pomerania, and Saxony-Anhalt were grouped as *northern states*. Conversely, the states of Hesse, Rhineland-Palatinate, Baden-Württemberg, Bavaria, Saarland, Saxony, and Thuringia were grouped as *southern states*. In order to examine the burden of disease in the regions of the former East Germany, the states of Brandenburg, Sachsen, Thuringia, Mecklenburg-West Pomerania, and Saxony-Anhalt were grouped as *eastern states*. Data from the formerly divided Berlin was represented separately. Data for each group were then further subdivided by age and gender, and further analysis was conducted accordingly. Further information on data collection and analysis can be found elsewhere [13].

### Software and statistical analysis

The IBM SPSS version 27 was used to conduct the descriptive statistical analysis [14]. Microsoft Excel for Office 365 was used to organize data and calculate incidence rates [15]. Tableau version 2020.1.2 was used to map the results on OpenStreetMaps and create incidence graphs [16]. Kaplan–Meier was used to calculate survival rates. The log-rank statistic was used to compare survival rates among different groups. A *p*-value of 0.05 or lower was considered significant for two-tailed tests. We calculated confidence intervals for 95% level [17, 18]. Directly standardized rates were calculated using methods mentioned elsewhere [17]. Annual percent change (APC) was calculated using the JoinPoint Software version 4.9.0 and using permutation test for selecting the model [19]. We also conducted a multivariate Cox regression analyses using “survminer” version 0.4.9 and “survival” version 3.2–10 packages in R software version 4.0.4 (2021–02-15)—the R Foundation for Statistical Computing, to estimate the hazard ratios of the influence of age at diagnosis, sex, topography, and geographical location on overall and cancer-specific survival.

## Results

### Patients’ characteristics

**Fig. 1:**
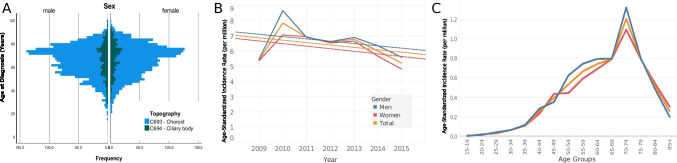
Distribution of patients. Age at diagnosis by topography by sex (**A**), age-standardized incidence rate per year (**B**), crude incidence rate (**C**), age-standardized incidence rate (**D**)

**Fig. 2:**
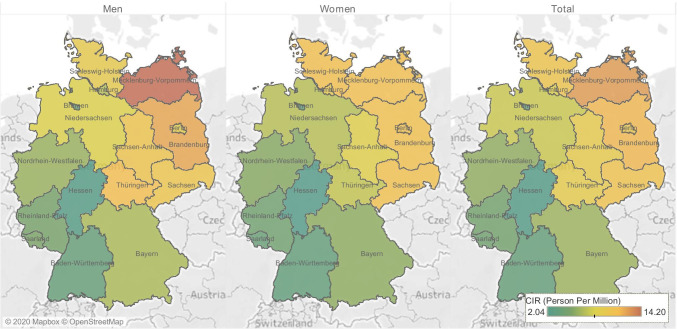
Crude incidence rates by state

Of the 3654 uveal melanoma diagnoses reviewed in this study, 3187 (87.2%) were choroidal melanomas (Table 1). Mean age at presentation was 65.41 (95% CI: 64.98–65.84) years of age, while the median stood at 67.5 (range = 83.5, interquartile range: 18.69) years. No significant differences in age of presentation were detected between men and women (t =  − 1.9, *p*-value = 0.055) or patients with choroidal versus ciliary body tumors (t =  − 1.7, p-value = 0.8) (Fig. 1, Supplementary Table 1). The overall age-standardized incidence of uveal melanoma was 6.41 per million (95% CI 6.21–6.62). The incidence of uveal melanoma was higher among men than women (6.67 (95% CI 6.37–6.98) vs. 6.16 (95% CI 5.88–6.45) per million). Northern states had higher crude incidence rates compared with southern states (7.65 (95% CI 7.33–7.97) vs 5.21 (95% CI 4.95–5.48) (Fig. 2, Supplementary Table 5). Mecklenburg-Vorpommern, a northern state, had the highest crude incidence rate among all German states (12.5 per million; 95% CI 10.5–14.7), while Hessen, a south-central state, had the lowest incidence rate (2.1 per million; 95% CI 1.7–2.6). Overall, incidences of uveal melanoma were higher in males than females (Fig. 1). The ASIR in males reached a peak of 1.32 per million in the 70–74 age group and in females in the same age group with ASIR of 1.1 per million. Of all patients, 55.7% were diagnosed at the ≥ 65 years of age, while only 7.5% were diagnosed at 15–45 years of age. The ASIR fluctuated over the years, reaching a peak in 2010 of 7.8 per million, and ended in a trough in 2015 at 5.2 per million. The overall trend showed a slight decreasing incidence with APC of − 2.8 (95% CI − 9.6–4.5, p-value = 0.359) (Fig. 1B). The details of the incidence are presented in Supplementary Tables 2, 3, 4, and 5.

**Table 1:**
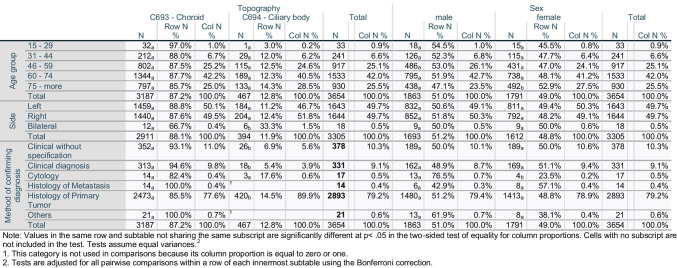
Patients Characteristics

### Tumor characteristics

Histological confirmation was reported for 79.2% of patients; diagnosis was determined solely clinically in another 19.4% (*n* = 709). Postoperative histopathological prefixes for TNM were added for 14% of patients (*n* = 541). TNM T was reported in 45% of all patients (*n* = 1252), including 9.7% (*n* = 354) as TNM Tx. Out of all patients reported with TNM T1-4, patients with T3 stood at 32.2% (*n* = 403), followed those with T2 (28.5%, *n* = 357) (Table 2). Further histological features are detailed in Table 2 and Supplementary Table 6. Ninety-eight tumors showed histological features of differentiation, including 74 (75.5%) well-differentiated tumors. Most of the tumors were reported as malignant histological subgroup NOS (Non-Otherwise Specified) melanomas (n = 3216, 88%), followed by spindle cell-NOS melanomas (5.4%, n = 197).

**Table 2:**
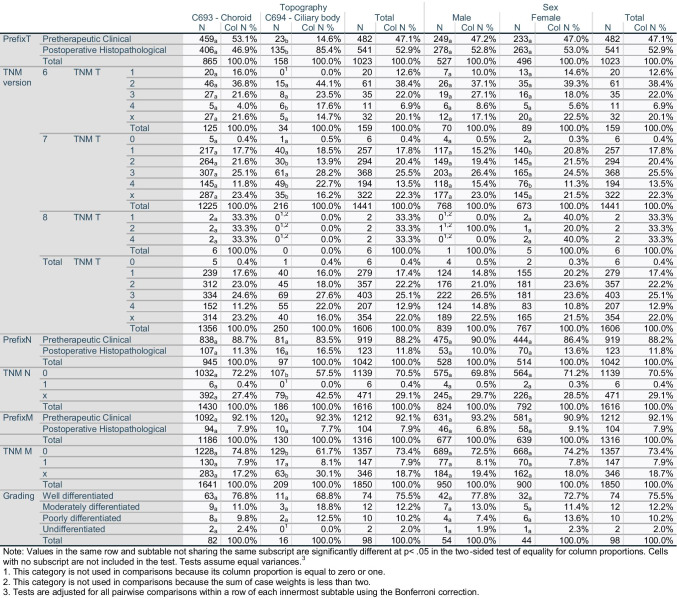
Stage of the disease

### Therapy

Records reported details of patient treatment for 253 (6.9%) patients. Of these, 79 (31.2%) only received radiation therapy, 60 (23.7%) underwent operations, and 39 (15.4%) were treated with both irradiation and a surgical intervention (Supplementary Table 7).

### Survival

**Fig. 3:**
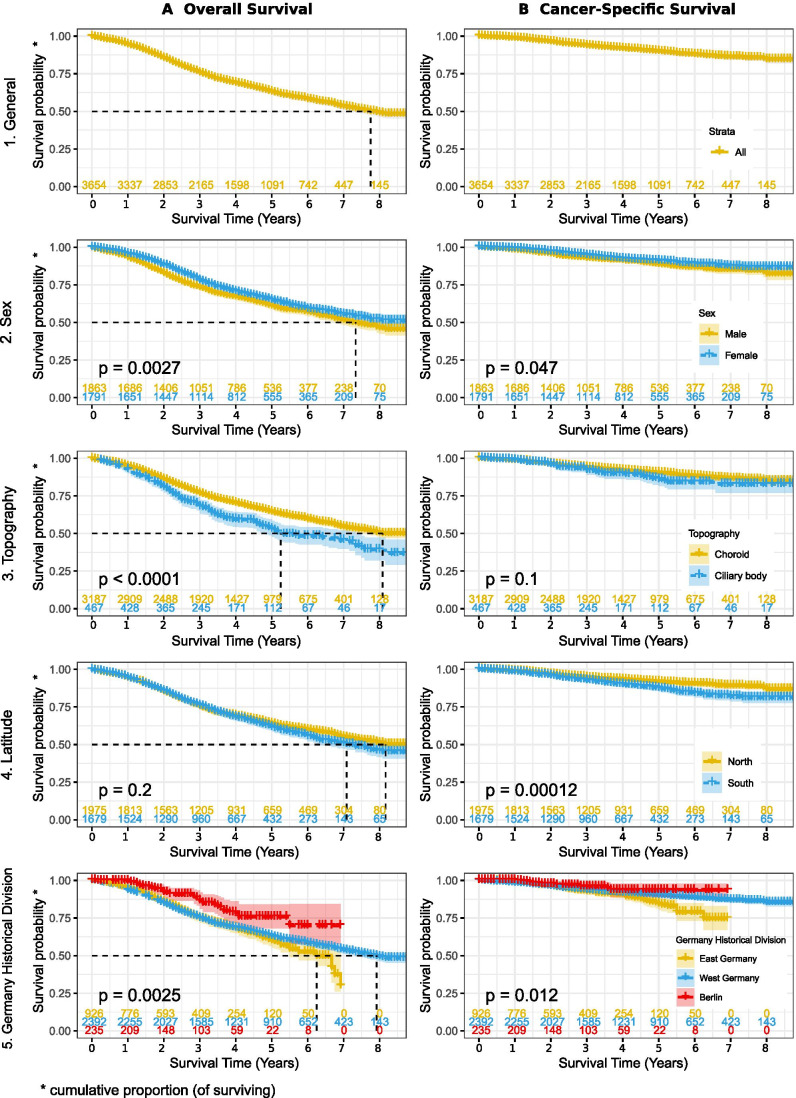
Overall and cancer-specific survival (A and B), by gender (2), topography (3), latitude (4), and Germany old segmentation scheme (5)

The 5-year cumulative overall survival rate stood at 47%, compared with an 84% cancer-specific survival rate. Women showed better overall survival compared with men (50% vs 45%, *p*-value < 0.001). Similarly, patients with choroidal melanomas showed better overall survival compared to those diagnosed with ciliary body melanomas (49% vs 37%, *p*-value < 0.001). Furthermore, women showed significantly better cancer-specific survival (85% compared with 82% in men, *p* = 0.03). Patients with choroidal melanomas showed a slightly better survival compared with those with ciliary body and iris (84% vs 80%, *p*-value = 0.168) (Fig. 3, Supplementary Figs. 1 and 2).

### Multivariate Cox regression

In our model, women (*p* = 0.001), younger age patients (*p* < 0.001), patients from Berlin (*p* = 0.001), and patients with choroidal tumors (*p* < 0.001) showed significantly higher overall survivals. In the cancer-specific survival model, those from Berlin (*p* < 0.001) showed significantly better survival (Supplementary Figs. 1 and 2).

## Discussion

Melanoma is the most common primary intraocular malignancy in adults, but it may affect orbital tissues at lower rates. Singh et al. reported the incidence in the USA to have stood at 5.1 per million [2]. This reported value nearly matched our findings with regard to central Germany. The age-specific incidence in the US population increased by age to reach a peak incidence in females at between 65 and 69 year of age and males at between 70 and 74 years of age. The previously mentioned US Surveillance, Epidemiology, and End Results Program “SEER” study showed a lower relative incidence of the disease among African Americans and Asian Americans. Unfortunately, the German national registry does not specify the ethnicity of patients. It would be worthwhile to conduct a study that examines ethnic variations of uveal melanomas, given the recent flow of refugees from both the Middle and Far East regions [20].

A study that included data from European cancer registries between 1983 and 1994, including Saarland (a German state, as aforementioned), reported incidence rates ranging from < 2 per million in southern Spain and Italy to > 8 in Norway and Denmark in the north, respectively [4]. Our study showed a similar distribution within the mentioned range but within the same country. The observed higher incidences in northern and eastern Germany compared with the southern and western regions of the country may be attributable to ethnic variations, with movement of populations with pigmented or less fair skin over generations from western and southern Europe on the one hand, and similar ethnic make-up in northern states as that of Nordic countries. Fair skin was associated with an increased incidence of uveal melanoma, possibly due to unusual exposure to sunlight [21]. However, the relationship to sunlight exposure is contradictory in literature [6, 22]. In this study, patterns of distribution of uveal melanoma were illustrated in maps, rather than point gradients, due to the fact that some German states extend over longitudes.

The highest reported population-based incidence worldwide was of Australian men. It stood at a rate of 10.9 per million, while the lowest population-based incidence was reported in Japan with 0.3 per million [23]. The mentioned high rate in Australia is may be attributable to its majority susceptible Caucasian White population of European origins. A previous study used two different methods to calculate incidences of uveal melanoma by integrating data from the population registry of Münster, a city in the federal state of Northrhine-Westphalia, with data from two case-controlled studies in that region. Two estimates, 2.3 and 8.6 per million, were reported [24]. We calculated an incidence of 5.7 per million for the same state, which is close to the mean of the previously mentioned two estimates from the earlier study. The earlier study by Virgili et al. also reported an incidence of 4.5 per million in the state of Saarland, approximating the incidence rate reported for the same state by our study (4.3 per million) [4].

Contrary to the increasing incidence of skin melanoma in the USA and other countries, uveal melanoma has shown varying annual incidence trends. For example, while Sweden has experienced a declining incidence of uveal melanoma over the years [22], Canada has had a small annual increase in incidence over time [25]. Our study has found a slight decline in incidence over time, a finding similar to that from north European countries. Our study showed varying incidence trends in each German state as well. We believe that further follow-up studies are vital for a better understanding of long-term trends.

Older age at diagnosis as well as diagnoses of ciliary body tumors was associated with worse overall survival. Previous studies have reported increased incidences of uveal melanomas as well as worse prognoses in older age groups [26, 27]. The better survival in younger age groups was attributed to the underlying histological features of uveal melanomas commonly associated with younger age. Other factors that may have influenced measured incidence rates may include social and psychological factors that drove certain patients to seek diagnosis and treatment. Furthermore, specific mutations, such as the SF3B1 mutation, were found to be associated with younger age, choroidal involvement, and a better prognosis[28]. On the other hand, BAP1 mutations were found to be associated with older age and worse prognosis. Notably, the lower survival rate of patients with ciliary body melanomas was found to be an independent prognostic factor for survival in a number of studies [26, 29, 30]. However, in our study, the cancer-specific survival was not significantly attributable to topography. This lower survival in the other studies was attributed to its higher rate of metastasis [31]. Risk of metastasis in these cases was reportedly related to tumor size, microvascular patterns, and monosomy 3 and 8q gain [32],

Better survival rates for women were a notable finding of our study. This, as a number of studies have suggested, could be attributed to women’s hormonal profile, varied genetic predispositions compared with men, and/or differences in occupational factors including amount of sun exposure [21, 33–35]. Other studies have attributed the differences to a tendency towards tumor extension, or to the involvement of ciliary body extensions [36]. We believe that those clinical aspects should be considered in parallel with the relevant molecular, genetic, and environmental backgrounds of each patient.

In general, the survival of patients from Berlin showed both higher overall and cancer-specific survivals, while those from both eastern and westerns states of Germany showed similar 5-year overall and cancer-specific survivals. On the other hand, graphs showed lower overall survivals after the period of 5 years, indicating a possible affection of health status on the long term, the effect of the age distribution between eastern and western states, or other factors that were not considered in this study. However, our analysis did not yield any differences in the distribution of uveal melanomas between different regions of Germany by age.

Most of the patients in our study were reported to have been diagnosed with the non-specified *8720/3 malignant melanoma* histological subtype. This may give an impression that pathological studies were either not carefully done or were erroneously reported by the registrars. On the contrary, this highlights the dependency on clinical diagnosis for the management of uveal melanomas, currently the cornerstone of the diagnosis of these tumors [37]. Clinical diagnosis has been reported to have had an accuracy of up to 99% [38]. This is clear for medium-sized to large melanomas, but small ones are often hard to diagnose. Moreover, this unspecified histological coding can be attributed to the success of clinical management, resulting in the unavailability of pathological samples. On the other hand, some authors have proposed that this was related to the increasing complexity of incidence calculation [39].

The low DCO (not presented) rate indicates a relatively high level of reliable data that can be used in further research. Both show the accuracy of the reporting system in the deliverance of accurate information at presentation or during management, allowing a chance for further follow-up. On the other hand, the discrepancy between the method of confirming diagnoses and histopathological staging indicates a gap in the reporting of the histopathological staging of uveal melanomas. Establishing a central cancer registry for uveal melanomas can help more accurately complementing the national general cancer registry.

This study is the first to report on the nationwide incidence of uveal melanomas in Germany. As with all registry-based studies, it has limitations. Some differences between states can be attributed to the degree of registry completion. Two of the southern registries (Hessen and Baden-Wuerttemberg) were founded in 2007–2009 and, therefore, are still being built up. Moreover, variations in the details reported by registries, including treatment and outcome, could have resulted from a lag time between diagnoses and reporting times. Furthermore, the small number of patients within some groups and states may result in inaccurate subgroup and trend analyses. Further efforts should be invested in follow-up studies and training ophthalmologists and cancer registrars on reporting uveal melanomas and other cancers as well.

In conclusion, the age-standardized incidence of uveal melanoma in Germany was 6.41 per million. Men showed higher standardized incidences and lower survivals than women. Patients with choroidal disease showed higher survivals than those with a ciliary body or iridial tumors. Patients from the former East Germany showed similar 5-year survival rates to those from the former West Germany.

## Supplementary Information

Below is the link to the electronic supplementary material.

Supplementary file1 (DOCX 2976 KB)

## Data Availability

Not applicable.
