# Supplementary material for: The epidemiology of uveal melanoma in Germany: a nationwide report of incidence and survival between 2009 and 2015
Source: Graefes Arch Clin Exp Ophthalmol. 2021 Oct 4;260(5):1723–31. doi: 10.1007/s00417-021-05317-7 (PMC9007804; doi:10.1007/s00417-021-05317-7)
Supplement: Supplementary file 1 — Supplementary file1 (DOCX 2976 KB) [file 417_2021_5317_MOESM1_ESM.docx]

Supplementary Tables

Supplementary Table 1: Age at diagnosis:

|  | | Age at Diagnosis | | | |  |  |  |
| --- | --- | --- | --- | --- | --- | --- | --- | --- |
|  |  | Mean | Standard Deviation | 95.0% Lower CL for Mean | 95.0% Upper CL for Mean | Median | Range | Interquartile Range |
| Sex | Male | 65.00 | 12.97 | 64.41 | 65.59 | 67 | 84 | 19 |
|  | Female | 65.84 | 13.39 | 65.22 | 66.46 | 68 | 78 | 19 |
|  | Total | 65.41 | 13.18 | 64.98 | 65.84 | 67 | 84 | 19 |
| Topography | C693 - Choroid | 65.27 | 13.12 | 64.81 | 65.72 | 67 | 83 | 19 |
|  | C694 - Ciliary body | 66.40 | 13.55 | 65.17 | 67.63 | 68 | 70 | 19 |

Supplementary Table 2: Crude Incidence Rate (CIR), per age group

|  | **2009** | | | **2010** | | | **2011** | | | **2012** | | | **2013** | | | **2014** | | | **2015** | | | **2009-2015** | | |
| --- | --- | --- | --- | --- | --- | --- | --- | --- | --- | --- | --- | --- | --- | --- | --- | --- | --- | --- | --- | --- | --- | --- | --- | --- |
| **Age** | M | F | Both | M | F | Both | M | F | Both | M | F | Both | M | F | Both | M | F | Both | M | F | Both | M | F | Both |
| **15-19** | 0.44 | 0.00 | 0.23 | 0.00 | 0.00 | 0.00 | 0.00 | 0.00 | 0.00 | 0.00 | 0.00 | 0.00 | 0.48 | 0.00 | 0.25 | 0.00 | 0.00 | 0.00 | 0.00 | 0.00 | 0.00 | 0.13 | 0.00 | 0.07 |
| **20-24** | 0.00 | 0.42 | 0.20 | 0.00 | 0.00 | 0.00 | 0.41 | 0.42 | 0.41 | 0.00 | 0.43 | 0.21 | 0.42 | 0.43 | 0.42 | 0.84 | 0.44 | 0.65 | 0.00 | 0.45 | 0.22 | 0.23 | 0.37 | 0.30 |
| **25-29** | 0.40 | 0.41 | 0.40 | 1.59 | 0.41 | 1.01 | 0.82 | 1.67 | 1.24 | 0.40 | 0.00 | 0.20 | 1.57 | 0.82 | 1.20 | 0.00 | 0.40 | 0.20 | 0.00 | 0.00 | 0.00 | 0.67 | 0.52 | 0.60 |
| **30-34** | 0.84 | 0.00 | 0.42 | 2.06 | 2.12 | 2.09 | 0.42 | 1.27 | 0.84 | 1.64 | 1.24 | 1.44 | 0.00 | 1.63 | 0.81 | 1.57 | 0.40 | 0.99 | 0.77 | 1.20 | 0.98 | 1.04 | 1.12 | 1.08 |
| **35-39** | 1.81 | 1.12 | 1.47 | 3.09 | 2.38 | 2.74 | 2.11 | 2.12 | 2.11 | 0.43 | 2.60 | 1.51 | 1.29 | 0.87 | 1.08 | 2.53 | 3.42 | 2.97 | 2.04 | 0.83 | 1.44 | 1.92 | 1.89 | 1.91 |
| **40-44** | 1.69 | 1.77 | 1.72 | 4.64 | 4.55 | 4.60 | 3.14 | 2.89 | 3.02 | 3.63 | 3.70 | 3.66 | 3.15 | 1.78 | 2.47 | 4.84 | 4.91 | 4.87 | 3.90 | 1.98 | 2.95 | 3.52 | 3.08 | 3.30 |
| **45-49** | 4.24 | 3.51 | 3.88 | 4.45 | 8.09 | 6.23 | 3.68 | 5.22 | 4.44 | 4.52 | 5.51 | 5.01 | 5.13 | 6.14 | 5.63 | 3.77 | 4.47 | 4.12 | 1.49 | 3.36 | 2.42 | 3.92 | 5.21 | 4.55 |
| **50-54** | 5.64 | 6.41 | 6.02 | 10.65 | 7.56 | 9.12 | 8.31 | 4.52 | 6.42 | 7.13 | 5.64 | 6.39 | 6.02 | 9.44 | 7.72 | 8.20 | 4.46 | 6.34 | 8.90 | 3.51 | 6.23 | 7.85 | 5.90 | 6.88 |
| **55-59** | 10.11 | 7.70 | 8.90 | 15.97 | 11.27 | 13.60 | 11.97 | 7.63 | 9.77 | 13.57 | 11.12 | 12.33 | 10.75 | 8.82 | 9.78 | 8.01 | 9.65 | 8.84 | 6.75 | 6.04 | 6.39 | 10.93 | 8.87 | 9.89 |
| **60-64** | 11.92 | 12.45 | 12.19 | 18.17 | 12.74 | 15.41 | 10.42 | 14.54 | 12.53 | 15.86 | 10.34 | 13.03 | 13.04 | 13.91 | 13.49 | 14.86 | 12.91 | 13.86 | 10.73 | 6.39 | 8.50 | 13.55 | 11.83 | 12.67 |
| **65-69** | 14.96 | 14.59 | 14.77 | 21.12 | 14.55 | 17.71 | 15.57 | 16.63 | 16.13 | 13.16 | 16.48 | 14.89 | 19.61 | 19.55 | 19.58 | 11.55 | 11.64 | 11.60 | 13.48 | 12.46 | 12.95 | 15.72 | 15.09 | 15.39 |
| **70-74** | 17.35 | 16.02 | 16.63 | 31.38 | 17.33 | 23.83 | 26.89 | 24.61 | 25.66 | 18.99 | 15.62 | 17.18 | 24.98 | 16.91 | 20.65 | 22.72 | 15.21 | 18.70 | 16.89 | 12.92 | 14.77 | 22.88 | 17.07 | 19.76 |
| **75-79** | 16.94 | 13.14 | 14.76 | 22.73 | 18.28 | 20.19 | 27.35 | 17.62 | 21.82 | 19.64 | 21.19 | 20.51 | 26.73 | 18.92 | 22.34 | 17.61 | 16.05 | 16.74 | 23.78 | 16.67 | 19.81 | 22.16 | 17.44 | 19.49 |
| **80-84** | 17.12 | 12.27 | 14.01 | 25.41 | 15.68 | 19.29 | 21.58 | 18.66 | 19.76 | 21.15 | 18.16 | 19.31 | 17.72 | 14.90 | 16.00 | 23.69 | 8.46 | 14.49 | 23.30 | 19.68 | 21.13 | 21.49 | 15.42 | 17.75 |
| **85 +** | 12.42 | 8.14 | 9.26 | 15.59 | 12.89 | 13.61 | 14.37 | 10.63 | 11.59 | 32.44 | 9.01 | 15.25 | 14.16 | 5.45 | 7.87 | 14.77 | 8.67 | 10.44 | 10.77 | 7.86 | 8.73 | 16.18 | 8.91 | 10.91 |
| **Total** | 5.33 | 5.27 | 5.30 | 8.55 | 7.01 | 7.77 | 6.92 | 6.96 | 6.94 | 6.74 | 6.59 | 6.66 | 7.04 | 6.78 | 6.91 | 6.50 | 5.76 | 6.12 | 5.82 | 4.94 | 5.37 | 6.70 | 6.18 | 6.44 |

|  |  | Men | 95% CI |  |  | Women | 95% CI |  |  | Both | 95% CI |  |
| --- | --- | --- | --- | --- | --- | --- | --- | --- | --- | --- | --- | --- |
| Age group | | CIR | Upper | Lower |  | CIR | Upper | Lower |  | CIR | Upper | Lower |
| 15-19 |  | 0.13 | 0.02 | 0.49 |  | 0.00 | 0.00 | 0.26 |  | 0.07 | 0.01 | 0.25 |
| 20-24 |  | 0.23 | 0.06 | 0.60 |  | 0.37 | 0.13 | 0.80 |  | 0.30 | 0.14 | 0.55 |
| 25-29 |  | 0.67 | 0.35 | 1.17 |  | 0.52 | 0.24 | 0.99 |  | 0.60 | 0.37 | 0.92 |
| 30-34 |  | 1.04 | 0.62 | 1.64 |  | 1.12 | 0.68 | 1.75 |  | 1.08 | 0.76 | 1.49 |
| 35-39 |  | 1.92 | 1.32 | 2.69 |  | 1.89 | 1.30 | 2.67 |  | 1.91 | 1.47 | 2.43 |
| 40-44 |  | 3.52 | 2.77 | 4.41 |  | 3.08 | 2.37 | 3.94 |  | 3.30 | 2.78 | 3.90 |
| 45-49 |  | 3.92 | 3.17 | 4.78 |  | 5.21 | 4.33 | 6.21 |  | 4.55 | 3.97 | 5.19 |
| 50-54 |  | 7.85 | 6.74 | 9.09 |  | 5.90 | 4.94 | 7.00 |  | 6.88 | 6.14 | 7.69 |
| 55-59 |  | 10.93 | 9.51 | 12.51 |  | 8.87 | 7.60 | 10.28 |  | 9.89 | 8.93 | 10.93 |
| 60-64 |  | 13.55 | 11.83 | 15.45 |  | 11.83 | 10.26 | 13.57 |  | 12.67 | 11.49 | 13.93 |
| 65-69 |  | 15.72 | 13.73 | 17.91 |  | 15.09 | 13.22 | 17.15 |  | 15.39 | 14.01 | 16.86 |
| 70-74 |  | 22.88 | 20.54 | 25.42 |  | 17.07 | 15.19 | 19.11 |  | 19.76 | 18.27 | 21.34 |
| 75-79 |  | 22.16 | 19.45 | 25.14 |  | 17.44 | 15.33 | 19.76 |  | 19.49 | 17.80 | 21.30 |
| 80-84 |  | 21.49 | 18.02 | 25.44 |  | 15.42 | 13.10 | 18.04 |  | 17.75 | 15.77 | 19.91 |
| 85+ |  | 16.18 | 12.41 | 20.74 |  | 8.91 | 7.17 | 10.96 |  | 10.91 | 9.25 | 12.79 |
| Total |  | 6.70 | 6.40 | 7.01 |  | 6.18 | 5.90 | 6.48 |  | 6.44 | 6.23 | 6.65 |

Supplementary Table 3: Age Standardized Incidence Rates, per million

|  | 2009 | | | 2010 | | | 2011 | | | 2012 | | | 2013 | | | 2014 | | | 2015 | | | 2009-2015 | | |
| --- | --- | --- | --- | --- | --- | --- | --- | --- | --- | --- | --- | --- | --- | --- | --- | --- | --- | --- | --- | --- | --- | --- | --- | --- |
| *Age* | M | F | Both | M | F | Both | M | F | Both | M | F | Both | M | F | Both | M | F | Both | M | F | Both | M | F | Both |
| *15-19* | 0.02 | 0.00 | 0.01 | 0.00 | 0.00 | 0.00 | 0.00 | 0.00 | 0.00 | 0.00 | 0.00 | 0.00 | 0.03 | 0.00 | 0.01 | 0.00 | 0.00 | 0.00 | 0.00 | 0.00 | 0.00 | 0.01 | 0.00 | 0.00 |
| *20-24* | 0.00 | 0.02 | 0.01 | 0.00 | 0.00 | 0.00 | 0.03 | 0.02 | 0.02 | 0.00 | 0.02 | 0.01 | 0.03 | 0.03 | 0.03 | 0.05 | 0.03 | 0.04 | 0.00 | 0.03 | 0.01 | 0.01 | 0.02 | 0.02 |
| *25-29* | 0.02 | 0.02 | 0.02 | 0.10 | 0.02 | 0.06 | 0.05 | 0.10 | 0.07 | 0.03 | 0.00 | 0.01 | 0.10 | 0.05 | 0.07 | 0.00 | 0.02 | 0.01 | 0.00 | 0.00 | 0.00 | 0.04 | 0.03 | 0.04 |
| *30-34* | 0.05 | 0.00 | 0.03 | 0.13 | 0.12 | 0.12 | 0.03 | 0.07 | 0.05 | 0.10 | 0.07 | 0.09 | 0.00 | 0.09 | 0.05 | 0.10 | 0.02 | 0.06 | 0.05 | 0.07 | 0.06 | 0.06 | 0.06 | 0.06 |
| *35-39* | 0.11 | 0.06 | 0.09 | 0.19 | 0.14 | 0.16 | 0.13 | 0.12 | 0.12 | 0.03 | 0.15 | 0.09 | 0.08 | 0.05 | 0.06 | 0.15 | 0.20 | 0.18 | 0.12 | 0.05 | 0.09 | 0.12 | 0.11 | 0.11 |
| *40-44* | 0.14 | 0.13 | 0.14 | 0.38 | 0.35 | 0.36 | 0.26 | 0.22 | 0.24 | 0.29 | 0.28 | 0.29 | 0.26 | 0.14 | 0.19 | 0.39 | 0.37 | 0.38 | 0.32 | 0.15 | 0.23 | 0.29 | 0.23 | 0.26 |
| *45-49* | 0.38 | 0.29 | 0.34 | 0.40 | 0.68 | 0.54 | 0.33 | 0.44 | 0.39 | 0.41 | 0.46 | 0.44 | 0.46 | 0.52 | 0.49 | 0.34 | 0.37 | 0.36 | 0.13 | 0.28 | 0.21 | 0.35 | 0.44 | 0.40 |
| *50-54* | 0.45 | 0.48 | 0.47 | 0.85 | 0.57 | 0.71 | 0.66 | 0.34 | 0.50 | 0.57 | 0.43 | 0.50 | 0.48 | 0.71 | 0.60 | 0.65 | 0.34 | 0.49 | 0.71 | 0.26 | 0.48 | 0.63 | 0.45 | 0.53 |
| *55-59* | 0.69 | 0.52 | 0.60 | 1.09 | 0.75 | 0.92 | 0.82 | 0.51 | 0.66 | 0.93 | 0.74 | 0.83 | 0.73 | 0.59 | 0.66 | 0.55 | 0.65 | 0.60 | 0.46 | 0.40 | 0.43 | 0.75 | 0.59 | 0.67 |
| *60-64* | 0.70 | 0.73 | 0.71 | 1.07 | 0.75 | 0.90 | 0.61 | 0.85 | 0.73 | 0.93 | 0.61 | 0.76 | 0.77 | 0.81 | 0.79 | 0.87 | 0.76 | 0.81 | 0.63 | 0.37 | 0.50 | 0.80 | 0.69 | 0.74 |
| *65-69* | 0.76 | 0.77 | 0.76 | 1.07 | 0.77 | 0.92 | 0.79 | 0.88 | 0.83 | 0.67 | 0.87 | 0.77 | 1.00 | 1.03 | 1.01 | 0.59 | 0.61 | 0.60 | 0.68 | 0.66 | 0.67 | 0.80 | 0.79 | 0.80 |
| *70-74* | 1.00 | 1.03 | 1.02 | 1.82 | 1.11 | 1.46 | 1.56 | 1.58 | 1.57 | 1.10 | 1.00 | 1.05 | 1.45 | 1.09 | 1.26 | 1.32 | 0.98 | 1.14 | 0.98 | 0.83 | 0.90 | 1.32 | 1.10 | 1.21 |
| *75-79* | 0.62 | 0.60 | 0.61 | 0.83 | 0.83 | 0.83 | 1.00 | 0.80 | 0.90 | 0.71 | 0.97 | 0.84 | 0.97 | 0.86 | 0.92 | 0.64 | 0.73 | 0.69 | 0.87 | 0.76 | 0.81 | 0.81 | 0.79 | 0.80 |
| *80-84* | 0.38 | 0.43 | 0.41 | 0.57 | 0.55 | 0.56 | 0.48 | 0.66 | 0.57 | 0.48 | 0.64 | 0.56 | 0.40 | 0.52 | 0.46 | 0.53 | 0.30 | 0.42 | 0.52 | 0.69 | 0.61 | 0.48 | 0.54 | 0.51 |
| *85+* | 0.15 | 0.28 | 0.22 | 0.19 | 0.44 | 0.32 | 0.18 | 0.36 | 0.27 | 0.40 | 0.31 | 0.36 | 0.18 | 0.19 | 0.19 | 0.18 | 0.30 | 0.25 | 0.13 | 0.27 | 0.21 | 0.20 | 0.31 | 0.26 |
| *100%* | 5.33 | 5.27 | 5.30 | 8.55 | 7.01 | 7.77 | 6.92 | 6.96 | 6.94 | 6.74 | 6.59 | 6.66 | 7.04 | 6.78 | 6.91 | 6.50 | 5.76 | 6.12 | 5.82 | 4.94 | 5.37 | 6.70 | 6.18 | 6.44 |
|  |  |  |  |  |  |  |  |  |  |  |  |  |  |  |  |  |  |  |  |  |  |  |  |  |
| *ASIR* | 5.49 | 5.38 | 5.43 | 8.68 | 7.09 | 7.86 | 6.92 | 6.96 | 6.94 | 6.64 | 6.55 | 6.60 | 6.92 | 6.67 | 6.80 | 6.37 | 5.67 | 6.03 | 5.61 | 4.83 | 5.22 | 6.67 | 6.16 | 6.41 |
| lower CI | 4.78 | 4.69 | 4.93 | 7.79 | 6.30 | 7.26 | 6.12 | 6.18 | 6.37 | 5.87 | 5.79 | 6.05 | 6.13 | 5.91 | 6.25 | 5.61 | 4.97 | 5.50 | 4.91 | 4.18 | 4.74 | 6.37 | 5.88 | 6.21 |
| Upper CI | 6.28 | 6.14 | 5.97 | 9.65 | 7.95 | 8.50 | 7.79 | 7.81 | 7.54 | 7.49 | 7.38 | 7.18 | 7.78 | 7.51 | 7.39 | 7.20 | 6.44 | 6.58 | 6.38 | 5.54 | 5.73 | 6.98 | 6.45 | 6.62 |

M: Male, F: Female, CI: 95% Confidence IntervalSupplementary Table 4: Cases per state per year

|  | 2015 | | | 2014 | | | 2013 | | | 2012 | | | | 2011 | | | | 2010 | | | | 2009 | | | | Total | | |
| --- | --- | --- | --- | --- | --- | --- | --- | --- | --- | --- | --- | --- | --- | --- | --- | --- | --- | --- | --- | --- | --- | --- | --- | --- | --- | --- | --- | --- |
|  | Total | M | F | Total | M | F | Total | M | F | Total | M | F | Total | | M | F | Total | | M | F | Total | | M | F | Total | | M | F |
| Schleswig-Holstein | 31 | 12 | 19 | 25 | 12 | 13 | 33 | 14 | 19 | 34 | 16 | 18 | 24 | | 11 | 13 | 31 | | 15 | 16 | 24 | | 11 | 13 | 202 | | 91 | 111 |
| Hamburg | 8 | 5 | 3 | 7 | 2 | 5 | 17 | 8 | 9 | 21 | 6 | 15 | 16 | | 7 | 9 | 24 | | 10 | 14 | 10 | | 4 | 6 | 103 | | 42 | 61 |
| Niedersachsen | 62 | 41 | 21 | 56 | 30 | 26 | 50 | 24 | 26 | 51 | 26 | 25 | 68 | | 31 | 37 | 83 | | 52 | 31 | 47 | | 18 | 29 | 417 | | 222 | 195 |
| Bremen | 2 | 0 | 2 | 1 | 1 | 0 | 4 | 1 | 3 | 2 | 0 | 2 | 4 | | 4 | 0 | 3 | | 2 | 1 | 1 | | 1 | 0 | 17 | | 9 | 8 |
| Nordrhein-Westfalen | 53 | 28 | 25 | 58 | 27 | 31 | 90 | 43 | 47 | 84 | 41 | 43 | 113 | | 58 | 55 | 223 | | 115 | 108 | 90 | | 52 | 38 | 711 | | 364 | 347 |
| Hessen | 8 | 6 | 2 | 11 | 4 | 7 | 7 | 3 | 4 | 18 | 10 | 8 | 12 | | 7 | 5 | 22 | | 11 | 11 | 13 | | 6 | 7 | 91 | | 47 | 44 |
| Rheinland-Pfalz | 12 | 5 | 7 | 19 | 12 | 7 | 27 | 15 | 12 | 17 | 8 | 9 | 18 | | 8 | 10 | 17 | | 9 | 8 | 17 | | 8 | 9 | 127 | | 65 | 62 |
| Baden-Württemberg | 40 | 22 | 18 | 43 | 25 | 18 | 53 | 25 | 28 | 39 | 18 | 21 | 41 | | 15 | 26 | 19 | | 13 | 6 | 18 | | 11 | 7 | 253 | | 129 | 124 |
| Bayern | 78 | 41 | 37 | 72 | 33 | 39 | 77 | 38 | 39 | 70 | 44 | 26 | 63 | | 34 | 29 | 88 | | 49 | 39 | 94 | | 47 | 47 | 542 | | 286 | 256 |
| Saarland | 1 | 0 | 1 | 6 | 3 | 3 | 9 | 6 | 3 | 2 | 2 | 0 | 5 | | 3 | 2 | 1 | | 0 | 1 | 6 | | 3 | 3 | 30 | | 17 | 13 |
| Berlin | 25 | 14 | 11 | 56 | 21 | 35 | 41 | 23 | 18 | 39 | 19 | 20 | 44 | | 16 | 28 | 19 | | 9 | 10 | 11 | | 2 | 9 | 235 | | 104 | 131 |
| Brandenburg | 17 | 10 | 7 | 34 | 22 | 12 | 35 | 23 | 12 | 34 | 13 | 21 | 32 | | 11 | 21 | 16 | | 9 | 7 | 29 | | 15 | 14 | 197 | | 103 | 94 |
| Mecklenburg-Vorpommern | 17 | 7 | 10 | 20 | 14 | 6 | 21 | 13 | 8 | 26 | 10 | 16 | 29 | | 19 | 10 | 13 | | 9 | 4 | 15 | | 7 | 8 | 141 | | 79 | 62 |
| Sachsen | 42 | 19 | 23 | 40 | 25 | 15 | 45 | 19 | 26 | 55 | 24 | 31 | 46 | | 24 | 22 | 38 | | 14 | 24 | 32 | | 16 | 16 | 298 | | 141 | 157 |
| Sachsen-Anhalt | 21 | 11 | 10 | 27 | 13 | 14 | 19 | 10 | 9 | 30 | 18 | 12 | 23 | | 13 | 10 | 18 | | 11 | 7 | 11 | | 4 | 7 | 149 | | 80 | 69 |
| Thüringen | 22 | 13 | 9 | 21 | 14 | 7 | 29 | 13 | 16 | 14 | 10 | 4 | 19 | | 10 | 9 | 20 | | 15 | 5 | 16 | | 9 | 7 | 141 | | 84 | 57 |
| Total | 439 | 234 | 205 | 496 | 258 | 238 | 557 | 278 | 279 | 536 | 265 | 271 | 557 | | 271 | 286 | 635 | | 343 | 292 | 434 | | 214 | 220 | 3654 | | 1863 | 1791 |

M: Male, F: Female

Supplementary Table 5: Crude Incidence Rates in the states, per year, per million

|  | 2015 | | | 2014 | | | 2013 | | | 2012 | | | 2011 | | | 2010 | | | 2009 | | | CIR | | |
| --- | --- | --- | --- | --- | --- | --- | --- | --- | --- | --- | --- | --- | --- | --- | --- | --- | --- | --- | --- | --- | --- | --- | --- | --- |
|  | Total | M | W | Total | M | W | Total | M | W | Total | M | W | Total | M | W | Total | M | W | Total | M | W | Total | M | W |
| Schleswig-Holstein | 10.8 | 8.6 | 13.0 | 8.8 | 8.7 | 9.0 | 11.7 | 10.2 | 13.2 | 12.1 | 11.7 | 12.5 | 8.6 | 8.1 | 9.0 | 10.9 | 10.8 | 11.1 | 8.5 | 7.9 | 9.0 | 10.2 | 9.4 | 11.0 |
| Hamburg | 4.5 | 5.7 | 3.3 | 4.0 | 2.3 | 5.5 | 9.7 | 9.4 | 10.0 | 12.1 | 7.1 | 16.8 | 9.3 | 8.4 | 10.2 | 13.4 | 11.4 | 15.3 | 5.6 | 4.6 | 6.6 | 8.4 | 7.0 | 9.7 |
| Niedersachsen | 7.8 | 10.5 | 5.2 | 7.2 | 7.8 | 6.5 | 6.4 | 6.3 | 6.6 | 6.6 | 6.8 | 6.3 | 8.7 | 8.1 | 9.3 | 10.5 | 13.4 | 7.7 | 5.9 | 4.6 | 7.2 | 7.6 | 8.2 | 7.0 |
| Bremen | 3.0 | 0.0 | 5.9 | 1.5 | 3.1 | 0.0 | 6.1 | 3.1 | 8.9 | 3.1 | 0.0 | 6.0 | 6.1 | 12.6 | 0.0 | 4.5 | 6.2 | 3.0 | 1.5 | 3.1 | 0.0 | 3.7 | 4.0 | 3.4 |
| Nordrhein-Westfalen | 3.0 | 3.2 | 2.7 | 3.3 | 3.1 | 3.4 | 5.1 | 5.0 | 5.2 | 4.8 | 4.8 | 4.8 | 6.4 | 6.8 | 6.1 | 12.5 | 13.2 | 11.8 | 5.0 | 6.0 | 4.2 | 5.7 | 6.0 | 5.5 |
| Hessen | 1.3 | 2.0 | 0.6 | 1.8 | 1.3 | 2.3 | 1.2 | 1.0 | 1.3 | 3.0 | 3.4 | 2.6 | 2.0 | 2.4 | 1.6 | 3.6 | 3.7 | 3.6 | 2.1 | 2.0 | 2.3 | 2.1 | 2.3 | 2.0 |
| Rheinland-Pfalz | 3.0 | 2.5 | 3.4 | 4.7 | 6.1 | 3.4 | 6.8 | 7.7 | 5.9 | 4.3 | 4.1 | 4.4 | 4.5 | 4.1 | 4.9 | 4.2 | 4.6 | 3.9 | 4.2 | 4.1 | 4.4 | 4.5 | 4.7 | 4.3 |
| Baden-Württemberg | 3.7 | 4.1 | 3.3 | 4.0 | 4.7 | 3.3 | 5.0 | 4.8 | 5.2 | 3.7 | 3.5 | 3.9 | 3.9 | 2.9 | 4.8 | 1.8 | 2.5 | 1.1 | 1.7 | 2.1 | 1.3 | 3.4 | 3.5 | 3.3 |
| Bayern | 6.1 | 6.5 | 5.7 | 5.7 | 5.3 | 6.1 | 6.1 | 6.1 | 6.1 | 5.6 | 7.2 | 4.1 | 5.1 | 5.6 | 4.6 | 7.0 | 8.0 | 6.1 | 7.5 | 7.7 | 7.4 | 6.1 | 6.6 | 5.7 |
| Saarland | 1.0 | 0.0 | 2.0 | 6.1 | 6.2 | 5.9 | 9.1 | 12.4 | 5.9 | 2.0 | 4.1 | 0.0 | 5.0 | 6.2 | 3.9 | 1.0 | 0.0 | 1.9 | 5.9 | 6.0 | 5.7 | 4.3 | 5.0 | 3.6 |
| Berlin | 7.1 | 8.1 | 6.1 | 16.1 | 12.4 | 19.7 | 12.0 | 13.8 | 10.3 | 11.6 | 11.6 | 11.6 | 13.2 | 9.9 | 16.4 | 5.5 | 5.3 | 5.7 | 3.2 | 1.2 | 5.1 | 9.8 | 8.9 | 10.7 |
| Brandenburg | 6.8 | 8.1 | 5.6 | 13.8 | 18.2 | 9.6 | 14.3 | 19.1 | 9.6 | 13.9 | 10.8 | 16.9 | 13.0 | 9.1 | 16.9 | 6.4 | 7.3 | 5.5 | 11.5 | 12.1 | 11.0 | 11.4 | 12.1 | 10.7 |
| Mecklenburg-Vorpommern | 10.5 | 8.8 | 12.3 | 12.5 | 17.8 | 7.4 | 13.2 | 16.5 | 9.9 | 16.2 | 12.7 | 19.7 | 18.0 | 24.0 | 12.3 | 7.9 | 11.1 | 4.8 | 9.1 | 8.6 | 9.6 | 12.5 | 14.2 | 10.8 |
| Sachsen | 10.3 | 9.4 | 11.1 | 9.9 | 12.6 | 7.3 | 11.1 | 9.6 | 12.6 | 13.6 | 12.1 | 15.0 | 11.3 | 12.1 | 10.6 | 9.2 | 6.9 | 11.3 | 7.7 | 7.8 | 7.5 | 10.4 | 10.1 | 10.8 |
| Sachsen-Anhalt | 9.4 | 9.9 | 8.8 | 12.1 | 11.9 | 12.3 | 8.5 | 9.1 | 7.9 | 13.3 | 16.3 | 10.4 | 10.1 | 11.7 | 8.6 | 7.7 | 9.6 | 5.9 | 4.7 | 3.5 | 5.8 | 9.4 | 10.3 | 8.5 |
| Thüringen | 10.1 | 12.1 | 8.2 | 9.7 | 13.2 | 6.4 | 13.4 | 12.2 | 14.6 | 6.5 | 9.4 | 3.6 | 8.7 | 9.3 | 8.1 | 8.9 | 13.6 | 4.4 | 7.1 | 8.1 | 6.1 | 9.2 | 11.1 | 7.4 |
| Total | 5.3 | 5.8 | 4.9 | 6.1 | 6.5 | 5.8 | 6.9 | 7.0 | 6.8 | 6.7 | 6.7 | 6.6 | 6.9 | 6.9 | 7.0 | 7.8 | 8.6 | 7.0 | 5.3 | 5.3 | 5.3 | 6.4 | 6.7 | 6.2 |

M: Male, F: Female, CIR: 95% Confidence Interval

Whole Period incidence with 95% confidence intervals

|  | CIR for Total | | | CIR for Male | | | CIR for Female | | |
| --- | --- | --- | --- | --- | --- | --- | --- | --- | --- |
| State | CIR | Lower CI | Upper CI | CIR | Lower CI | Upper CI | CIR | Lower CI | Upper CI |
| Schleswig-Holstein | 10.21 | 8.85 | 11.72 | 9.42 | 7.59 | 11.57 | 10.96 | 9.02 | 13.20 |
| Hamburg | 8.37 | 6.83 | 10.15 | 7.01 | 5.05 | 9.47 | 9.66 | 7.39 | 12.40 |
| Niedersachsen | 7.59 | 6.88 | 8.35 | 8.23 | 7.18 | 9.38 | 6.97 | 6.03 | 8.03 |
| Bremen | 3.68 | 2.14 | 5.89 | 3.99 | 1.82 | 7.57 | 3.39 | 1.46 | 6.67 |
| Nordrhein-Westfalen | 5.74 | 5.32 | 6.18 | 6.02 | 5.42 | 6.68 | 5.47 | 4.91 | 6.07 |
| Hessen | 2.14 | 1.73 | 2.63 | 2.26 | 1.66 | 3.00 | 2.03 | 1.48 | 2.73 |
| Rheinland-Pfalz | 4.53 | 3.77 | 5.39 | 4.72 | 3.64 | 6.02 | 4.34 | 3.33 | 5.56 |
| Baden-Württemberg | 3.38 | 2.98 | 3.83 | 3.50 | 2.92 | 4.16 | 3.27 | 2.72 | 3.89 |
| Bayern | 6.15 | 5.64 | 6.69 | 6.60 | 5.86 | 7.41 | 5.71 | 5.03 | 6.46 |
| Saarland | 4.28 | 2.89 | 6.11 | 4.98 | 2.90 | 7.97 | 3.62 | 1.92 | 6.19 |
| Berlin | 9.79 | 8.57 | 11.12 | 8.86 | 7.24 | 10.74 | 10.67 | 8.92 | 12.66 |
| Brandenburg | 11.38 | 9.85 | 13.09 | 12.06 | 9.84 | 14.63 | 10.72 | 8.66 | 13.12 |
| Mecklenburg-Vorpommern | 12.47 | 10.50 | 14.70 | 14.15 | 11.20 | 17.63 | 10.83 | 8.30 | 13.88 |
| Sachsen | 10.42 | 9.27 | 11.67 | 10.07 | 8.47 | 11.87 | 10.75 | 9.14 | 12.57 |
| Sachsen-Anhalt | 9.34 | 7.90 | 10.97 | 10.23 | 8.12 | 12.74 | 8.48 | 6.60 | 10.73 |
| Thüringen | 9.20 | 7.74 | 10.85 | 11.11 | 8.87 | 13.76 | 7.34 | 5.56 | 9.51 |
| Total | 6.43 | 6.22 | 6.64 | 6.68 | 6.38 | 6.99 | 6.18 | 5.90 | 6.47 |
| Northern | 7.65 | 7.33 | 7.97 | 7.87 | 7.41 | 8.35 | 7.43 | 6.99 | 7.88 |
| Southern | 5.21 | 4.95 | 5.48 | 5.50 | 5.12 | 5.91 | 4.93 | 4.57 | 5.30 |

Supplementary Table 6: Histology reported in the registry

|  | | Topography | | | | | | | | | | | | | Sex | | | | | | | | | | | | |
| --- | --- | --- | --- | --- | --- | --- | --- | --- | --- | --- | --- | --- | --- | --- | --- | --- | --- | --- | --- | --- | --- | --- | --- | --- | --- | --- | --- |
|  |  | C693 - Choroid | | | | C694 - Ciliary body | | | | Total | | | | male | | | | | female | | | | Total | | | |  |
|  |  | N | Row N % | Col N % | N | | Row N % | Col N % | N | |  | Col N % | N | | | Row N % | Col N % | N | | Row N % | Col N % | N | |  | Col N % |  |  |
| Morphology according to ICD-O-3 | 8720 Malignant melanoma, NOS | 2830 | 88.0% | 88.8% | 386 | | 12.0% | 82.7% | 3216 | |  | 88.0% | 1620 | | | 50.4% | 87.0% | 1596 | | 49.6% | 89.1% | 3216 | |  | 88.0% |  |  |
|  | 8721 Nodular melanoma | 10 | 76.9% | 0.3% | 3 | | 23.1% | 0.6% | 13 | |  | 0.4% | 8 | | | 61.5% | 0.4% | 5 | | 38.5% | 0.3% | 13 | |  | 0.4% |  |  |
|  | 8730 Nonpigmented nevus | 47 | 90.4% | 1.5% | 5 | | 9.6% | 1.1% | 52 | |  | 1.4% | 28 | | | 53.8% | 1.5% | 24 | | 46.2% | 1.3% | 52 | |  | 1.4% |  |  |
|  | 8770 Mixed epithelioid and spindle cell melanoma | 25 | 69.4% | 0.8% | 11 | | 30.6% | 2.4% | 36 | |  | 1.0% | 16 | | | 44.4% | 0.9% | 20 | | 55.6% | 1.1% | 36 | |  | 1.0% |  |  |
|  | 8771 Epithelioid cell melanoma | 30 | 88.2% | 0.9% | 4 | | 11.8% | 0.9% | 34 | |  | 0.9% | 14 | | | 41.2% | 0.8% | 20 | | 58.8% | 1.1% | 34 | |  | 0.9% |  |  |
|  | 8772 Spindle cell melanoma, NOS | 163 | 82.7% | 5.1% | 34 | | 17.3% | 7.3% | 197 | |  | 5.4% | 112 | | | 56.9% | 6.0% | 85 | | 43.1% | 4.7% | 197 | |  | 5.4% |  |  |
|  | 8773 Spindle cell melanoma, type A | 22 | 81.5% | 0.7% | 5 | | 18.5% | 1.1% | 27 | |  | 0.7% | 15 | | | 55.6% | 0.8% | 12 | | 44.4% | 0.7% | 27 | |  | 0.7% |  |  |
|  | 8774 Spindle cell melanoma, type B | 48 | 71.6% | 1.5% | 19 | | 28.4% | 4.1% | 67 | |  | 1.8% | 40 | | | 59.7% | 2.1% | 27 | | 40.3% | 1.5% | 67 | |  | 1.8% |  |  |
|  | Others | 12 | 100% | 0.2% |  | |  |  | 12 | |  | 0.33% | 10 | | | 83.33% | 0.70% | 2 | | 16.67% | 0.21% | 12 | |  | 0.33% |  |  |
|  | Total | 3187 | 87.2% | 100.0% | 467 | | 12.8% | 100.0% | 3654 | |  | 100.0% | 1863 | | | 51.0% | 100.0% | 1791 | | 49.0% | 100.0% | 3654 | |  | 100.0% |  |  |

Supplementary Table 7: Treatment*

|  | | Topography | | | | | | | | | | | Sex | | | | | | | | | | | | |  |
| --- | --- | --- | --- | --- | --- | --- | --- | --- | --- | --- | --- | --- | --- | --- | --- | --- | --- | --- | --- | --- | --- | --- | --- | --- | --- | --- |
|  |  | C693 - Choroid | | | C694 - Ciliary body | | | | Total | | | | male | | | | female | | | | Total | | | |  |  |
|  |  | N |  | Col N % | N |  | Col N % | N | |  | Col N % | N | |  | Col N % | N | |  | Col N % | N | |  | Col N % |  |  |  |
| Treatment | OP, Chemo, Rad, and Others | 1_a_ |  | 0.4% | ^1^ |  |  | 1 | |  | 0.4% | ^1^ | |  |  | 1_a_ | |  | 0.9% | 1 | |  | 0.4% |  |  |  |
|  | OP, Chemo, and others | 6_a_ |  | 2.6% | 1_a_ |  | 3.8% | 7 | |  | 2.8% | 5_a_ | |  | 3.6% | 2_a_ | |  | 1.7% | 7 | |  | 2.8% |  |  |  |
|  | OP and Rad | 36_a_ |  | 15.9% | 3_a_ |  | 11.5% | 39 | |  | 15.4% | 22_a_ | |  | 16.1% | 17_a_ | |  | 14.7% | 39 | |  | 15.4% |  |  |  |
|  | OP, Chemo and Others | 3_a_ |  | 1.3% | ^1^ |  |  | 3 | |  | 1.2% | 2_a_ | |  | 1.5% | 1_a_ | |  | 0.9% | 3 | |  | 1.2% |  |  |  |
|  | OP and Chemo | 1_a_ |  | 0.4% | ^1^ |  |  | 1 | |  | 0.4% | 1_a_ | |  | 0.7% | ^1^ | |  |  | 1 | |  | 0.4% |  |  |  |
|  | OP and Others | 13_a_ |  | 5.7% | 2_a_ |  | 7.7% | 15 | |  | 5.9% | 11_a_ | |  | 8.0% | 4_a_ | |  | 3.4% | 15 | |  | 5.9% |  |  |  |
|  | Only OP | 50_a_ |  | 22.0% | 10_a_ |  | 38.5% | 60 | |  | 23.7% | 32_a_ | |  | 23.4% | 28_a_ | |  | 24.1% | 60 | |  | 23.7% |  |  |  |
|  | Rad, Chemo and Others | 2_a_ |  | 0.9% | ^1^ |  |  | 2 | |  | 0.8% | ^1^ | |  |  | 2_a_ | |  | 1.7% | 2 | |  | 0.8% |  |  |  |
|  | Rad and Chemo | 2_a_ |  | 0.9% | ^1^ |  |  | 2 | |  | 0.8% | 1_a_ | |  | 0.7% | 1_a_ | |  | 0.9% | 2 | |  | 0.8% |  |  |  |
|  | Rad and Others | 11_a_ |  | 4.8% | 1_a_ |  | 3.8% | 12 | |  | 4.7% | 5_a_ | |  | 3.6% | 7_a_ | |  | 6.0% | 12 | |  | 4.7% |  |  |  |
|  | Only Rad | 73_a_ |  | 32.2% | 6_a_ |  | 23.1% | **79** | |  | **31.2%** | 40_a_ | |  | 29.2% | 39_a_ | |  | 33.6% | 79 | |  | 31.2% |  |  |  |
|  | Chemo and Others | 1_a_ |  | 0.4% | ^1^ |  |  | 1 | |  | 0.4% | ^1^ | |  |  | 1_a_ | |  | 0.9% | 1 | |  | 0.4% |  |  |  |
|  | Only Chemo | 1_a_ |  | 0.4% | 1_a_ |  | 3.8% | 2 | |  | 0.8% | 2_a_ | |  | 1.5% | ^1^ | |  |  | 2 | |  | 0.8% |  |  |  |
|  | Only others | 10_a_ |  | 4.4% | 1_a_ |  | 3.8% | 11 | |  | 4.3% | 6_a_ | |  | 4.4% | 5_a_ | |  | 4.3% | 11 | |  | 4.3% |  |  |  |
|  | None | 17_a_ |  | 7.5% | 1_a_ |  | 3.8% | 18 | |  | 7.1% | 10_a_ | |  | 7.3% | 8_a_ | |  | 6.9% | 18 | |  | 7.1% |  |  |  |
|  | Total | 227 |  | 100.0% | 26 |  | 100.0% | **253** | |  | 100.0% | 137 | |  | 100.0% | 116 | |  | 100.0% | 253 | |  | 100.0% |  |  |  |
| *The treatment details represent the treatment at the point of data collection, it may not represent the whole population.  Note: Values in the same row and subtable not sharing the same subscript are significantly different at p< .05 in the two-sided test of equality for column proportions. Cells with no subscript are not included in the test. Tests assume equal variances.^2^ | | | | | | | | | | | | | | | | | | | | | | | | | | |
| 1. This category is not used in comparisons because its column proportion is equal to zero or one. | | | | | | | | | | | | | | | | | | | | | | | | | | |

| Supplementary Figure 1: Hazard Ratios of Overall Survival Analysis: Cox-Regression Analysis and testing the proportional hazards assumption | |
| --- | --- |
| A. Overall survival with assumption of latitude | B. Overall survival without assumption of latitude |
| 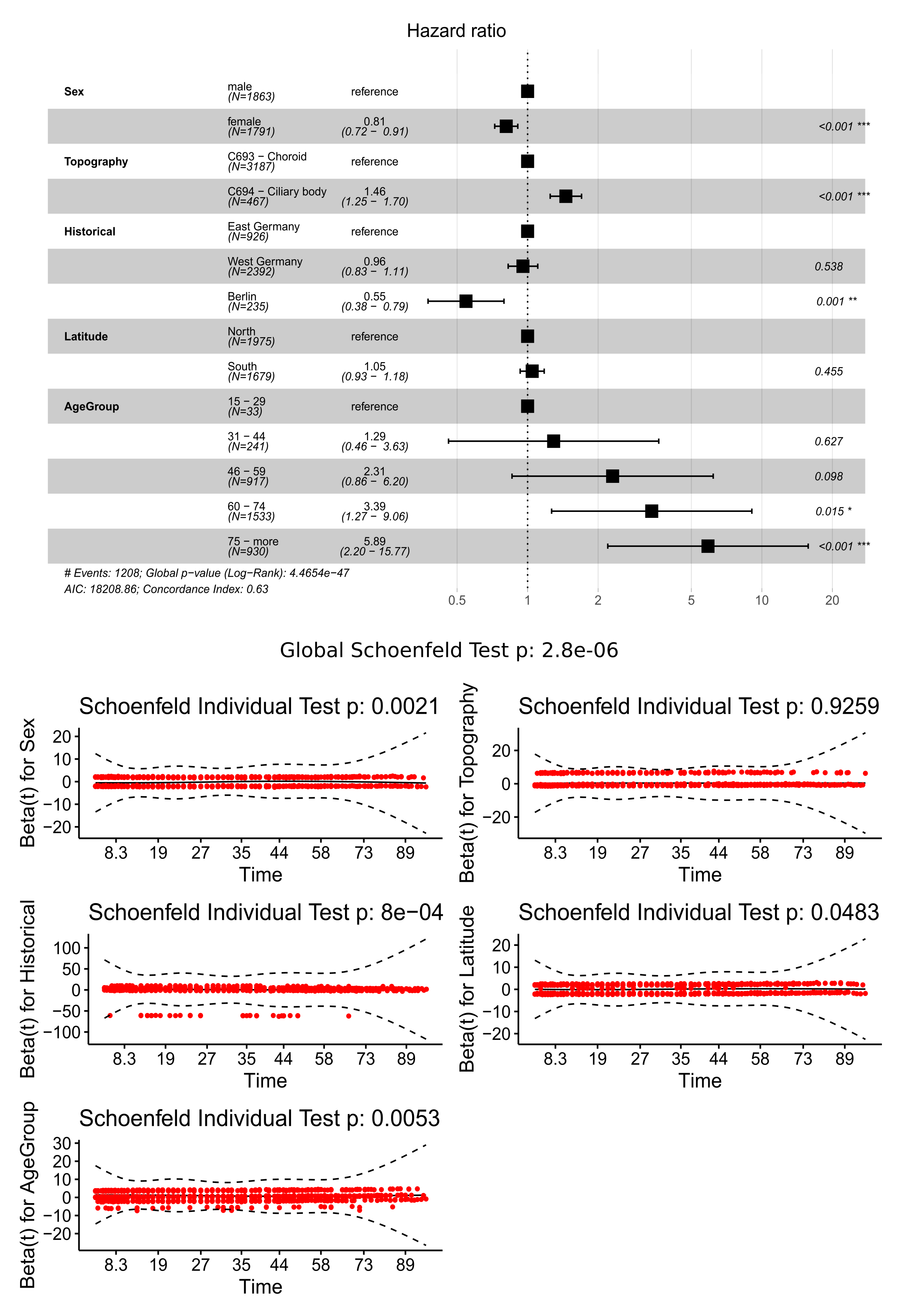 | 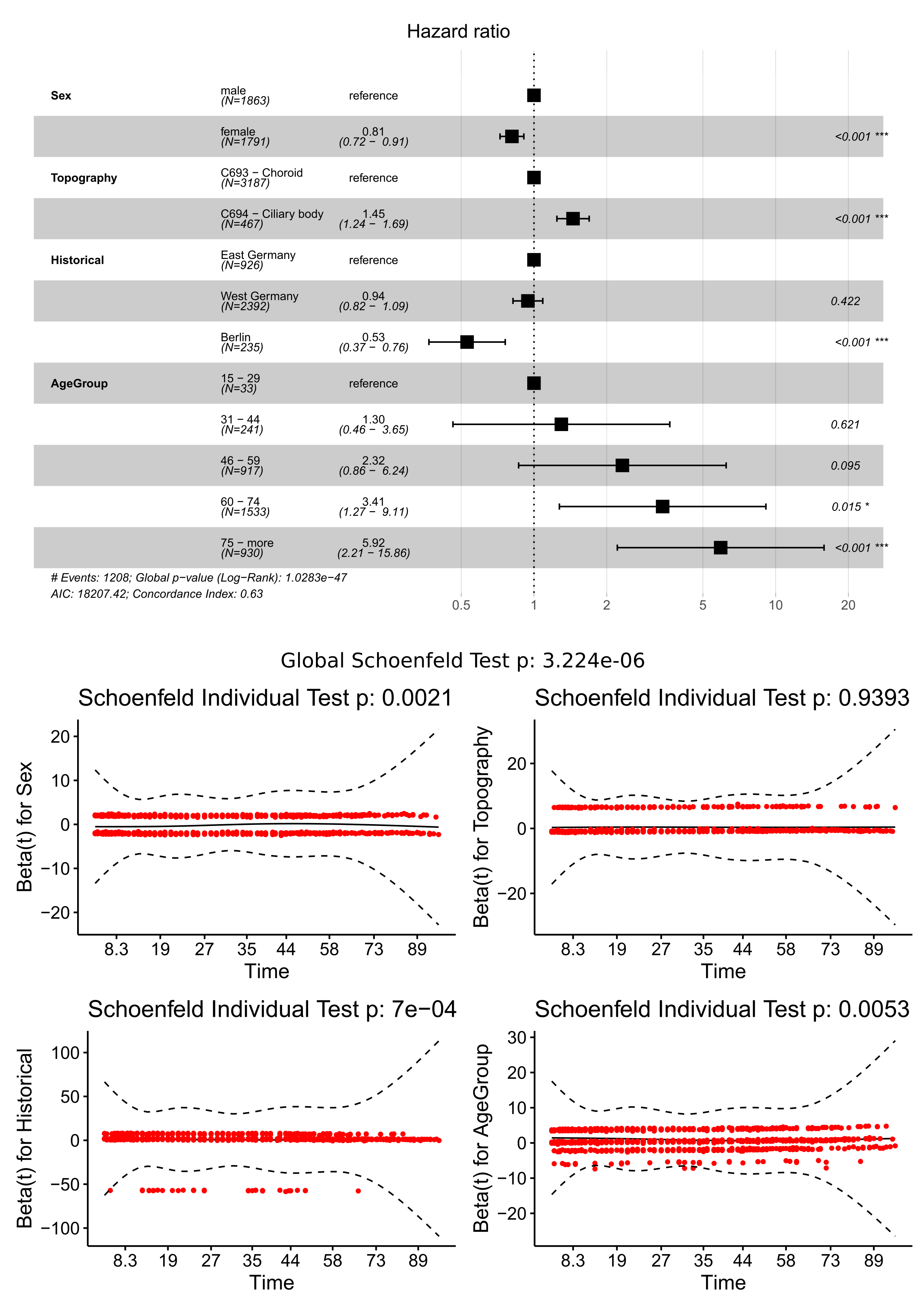 |
| Supplementary Figure 2: Hazard Ratios of Cancer-Specific Survival Analysis: Cox-Regression Analysis and testing the proportional hazards assumption | |
| A. Cancer-Specific survival with assumption of latitude | B. Cancer-Specific survival without assumption of latitude |
| 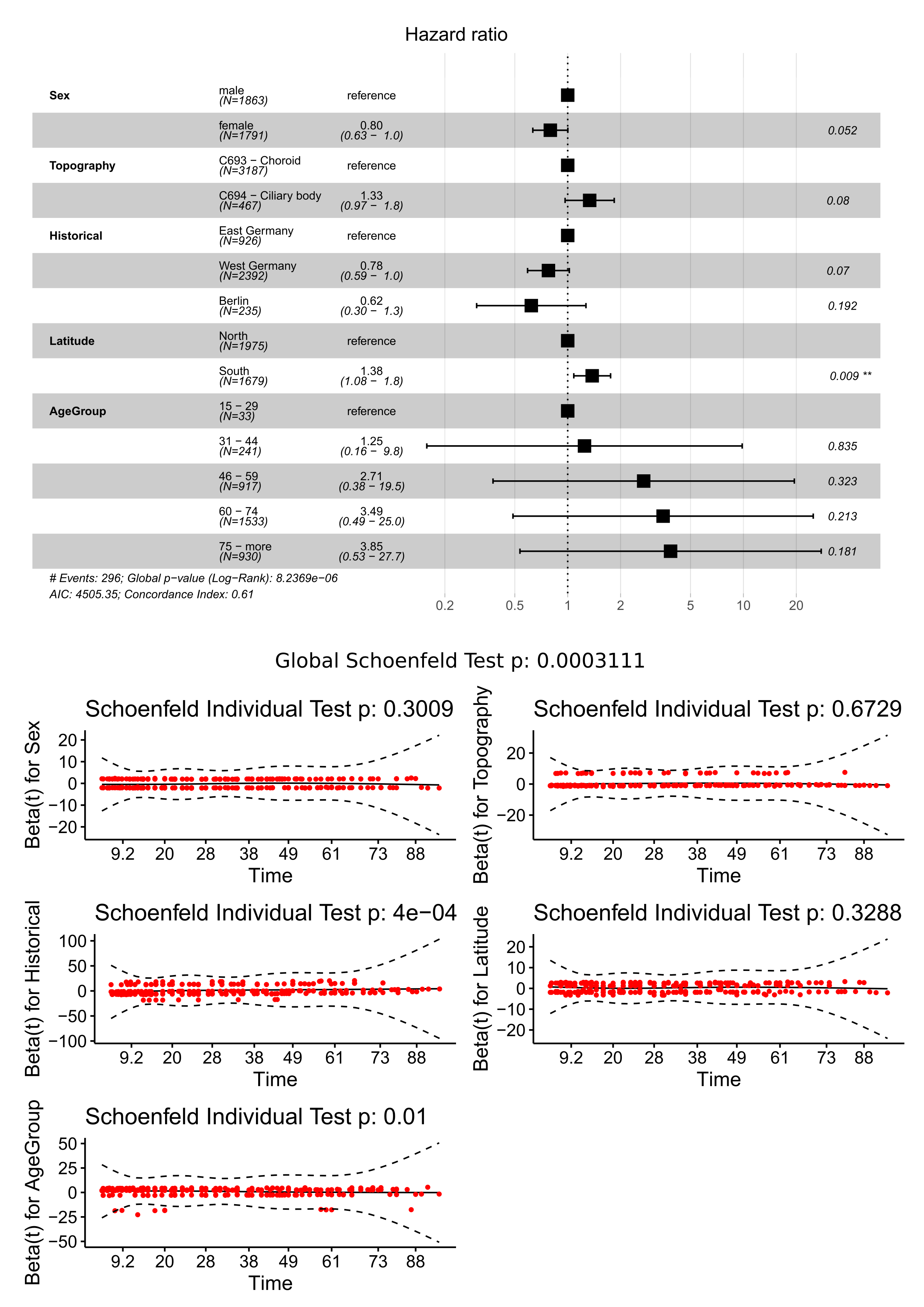 | 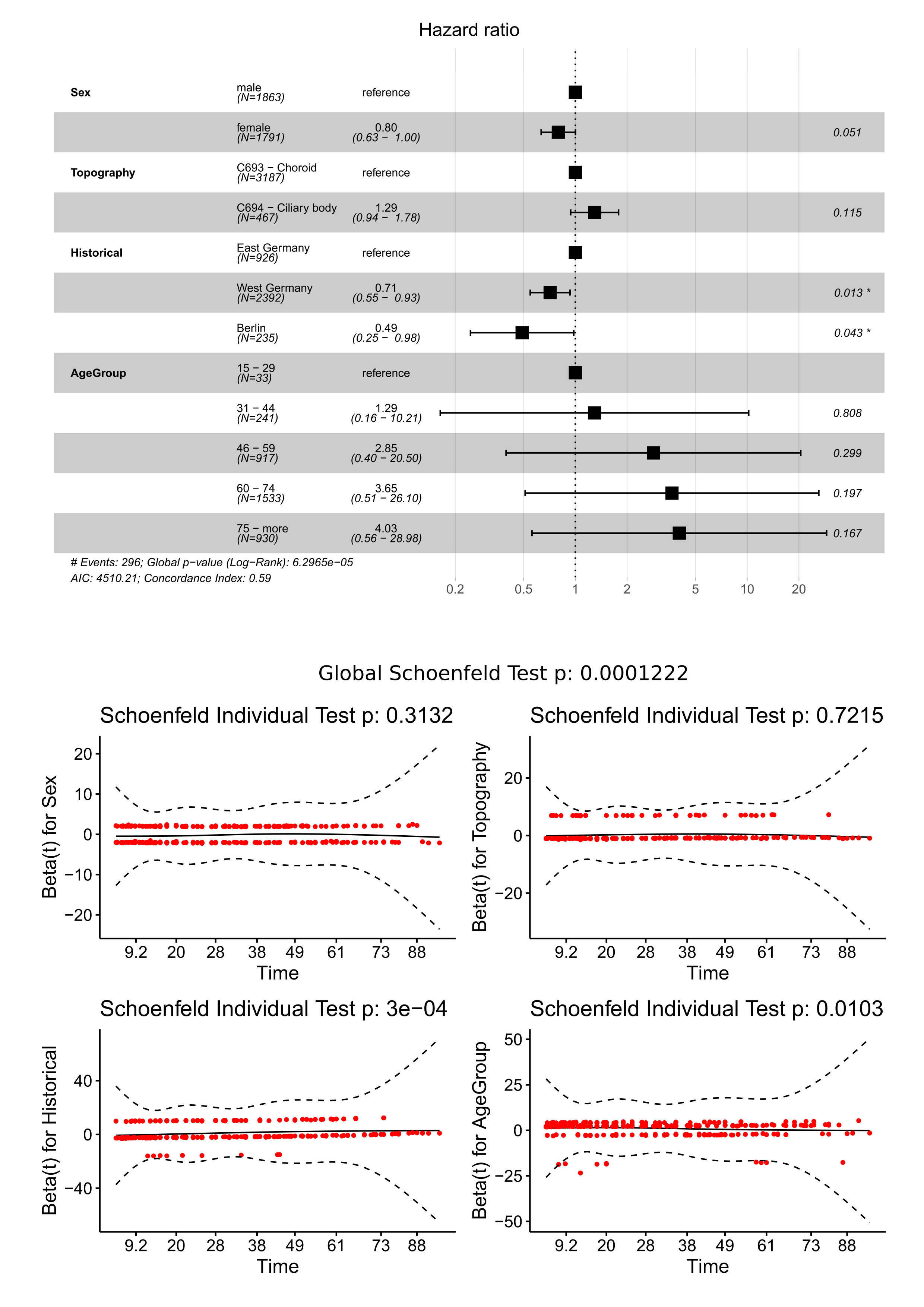 |
